# The Assessment of Viability of *M. Tuberculosis* after Exposure to CPC Using Different Methods

**DOI:** 10.1155/2014/564109

**Published:** 2014-01-28

**Authors:** Gomathi Sekar, R. Lakshmi, N. Selvakumar

**Affiliations:** Department of Bacteriology, National Institute for Research in Tuberculosis (ICMR), Chetput, Chennai, Tamil Nadu 600031, India

## Abstract

*Settings*. National Institute for Research in Tuberculosis, Chennai. *Objective*. To assess the proportion of metabolically active cells of *Mycobacterium tuberculosis* after exposed to CPC using FDA-EB vital staining and viable counts on LJ medium. Mycolic acid content in *M. tuberculosis* after exposure to CPC was estimated using HPLC. *Methods*. Clinical isolates of *M. tuberculosis* and standard reference strain *M. tuberculosis* H37Rv were used for FDA-EB, viable count, and HPLC. *Results*. FDA/EB consistently stained 70–90% of log phase cells as green and the remaining cells as red-orange. After CPC treatment, 65–70% of the cells stained red-orange. The viability counts were comparable to 0-day controls. Synthesis of mycolic acids in mycobacteria was reduced when exposed to CPC using HPLC due to the decreased metabolic activity of the organisms. *Conclusion*. The cells are metabolically inactive during storage with CPC but these cells grew well on LJ medium after removal of CPC. The viability of *M. tuberculosis* was maintained in CPC with minimal reduction. Mycolic acid content was reduced if the cells of *M. tuberculosis* were treated with CPC for 7 days. All the above findings provide yet another evidence for the damage of cell wall of *M. tuberculosis*.

## 1. Introduction

Tuberculosis caused by *Mycobacterium tuberculosis *is a public health problem which has increased in importance during the last 12 years, due in part to the increasing number of cases caused by the association of acquired immunodeficiency syndrome (AIDS) and the appearance of multiple drug-resistant strains [1–3]. Other mycobacteria which are often indistinguishable from tuberculosis have also increased. Sputum samples collected from remote areas to central laboratories were transported in CPC. Use of cetylpyridinium chloride (CPC) as transport medium for the recovery of *M. tuberculosis *is established and accepted procedure. CPC plays a dual role as liquefaction as well as decontamination agent. It is usually recommended to allow minimum exposure of sputum specimen with decontamination agents (e.g., NaOH in modified Petroff's method) as they tend to reduce the viability of the bacilli. But sputum specimens collected with CPC are processed after 5–7 days of collection. The cell wall integrity of *M. tuberculosis *was disturbed during transportation but when the CPC was washed from the sample, the cell wall regained and grew normally on LJ medium (publication in process). Mode of action of CPC on *M. tuberculosis * and its recovery from sputum is not clearly documented. Moreover, liquid CPC has some usage constraint. Hence the study was carried out to check and document the action of CPC on the viability of *M. tuberculosis *and the use of powder form of CPC as alternative to liquid form of CPC.

Attempt was made to use powder form of CPC (P-CPC) replacing the use of liquid form of CPC (L-CPC) used at a concentration of 7.6 mg/mL of sputum. In order to detect the viability of mycobacteria in CPC, collected sputum samples were stored at ambient temperature for up to 7 days and checked for viability at every time point. To assess the viable counts (VC) on LJ medium; the proportion of metabolically active cells of *M. tuberculosis *after exposure to CPC using FDA-EB vital staining method and the mycolic content of *M. tuberculosis *is also done.

## 2. Methods

Four clinical isolates and standard reference strain *M. tuberculosis *(H37Rv) were used for the study. Log phase cultures were used for the study after subculturing from original LJ slopes. Middlebrook 7H9 broth supplemented with 10% bovine serum albumin and 0.5% Tween 80 was prepared.

### 2.1. Preparation of Single Cell Suspension

The 7-day-old cultures were suspended and vortexed in 7H9 medium. After incubation for 5 days, turbidity of the culture was adjusted to McFarland 4 units and centrifuged (Eppendorf AG, 22231 Hamberg, Germany) at 14,000 rcf for 10 minutes. The pellet was resuspended in 6 mL of phosphate buffered saline (PBS) buffer and 4 aliquots of 1.5 mL each were made. The first aliquot (on zero day) was aseptically transferred to a 10 mL syringe and filtered through a 5 *μ*m membrane filter (Sartorius) to obtain single cell suspension.

### 2.2. FDA-EB Staining

One hundred microliter of single cell suspension was mixed with 100 *μ*L of freshly prepared FDA-EB staining solution and incubated for 15 min in dark at room temperature. A wet mount was prepared and sealed to prevent evaporation and observed under fluorescent microscope at a magnification of 450x (Olympus BX 40, Japan) with blue filter. A total of 200 cells were counted in duplicates and differentiated on the basis of colour.

### 2.3. Viable Count

Viable count of single cell suspension was set up on LJ medium by the usual procedure described above. The second aliquot (7-day-old culture suspension) was left at ambient conditions for 7 days. The third and fourth aliquotes were mixed with powder form of CPC. Currently, CPC is used in liquid form at 75 mg/5 ml concentration. Since the dilution of CPC is nullified when using powder form, the concentration of CPC was reduced to 50% (38 mg/5 mL). All the aliquots were incubated at ambient conditions for 7 days. On day 7, the aliquots were processed for FDA-EB staining [4, 5] and viable count was set up.

### 2.4. HPLC

Mycolic acids were extraction was carried out as per CDC protocol [6]. Six clinical isolates and one standard strain (H37Rv) were selected and subcultured on LJ medium to get fresh log phase growth. Clinical isolates of *M. tuberculosis *used for mycolic acid analysis were different from those used in viable count. Two loopfuls of the fresh culture was suspended in two milliliters of distilled water, vortexed, and aliquoted into two equal parts. The first aliquot was processed for mycolic acid extraction. The second aliquot was mixed with 7.6 mg of CPC and kept at ambient conditions. On the 7th day, samples were washed with distilled water and processed for HPLC analysis as per the procedure described by Viader-Salvadó et al. [7].

### 2.5. HPLC Analysis

Chromatography was performed using HPLC system (Agilent 1100 series, Agilent technology Ltd., Germany) with UV detector set at 254 nm to 350 nm and data capture unit controlled by software inbuilt in the Agilent HPLC instrument. Mycolic acid was separated using reverse phase C18 column (150 mm × 4.6 mm) packed with 5 micron particles (Purospher, Merck Ltd). A solvent gradient system described by Hagen and Thompson was followed with minor modification [8]. After injection, the initial solvent mixture (methanol: methylene chloride—70 : 30) was maintained for one minute at a constant flow rate of 2.0 mL/minute. Over the next 15 minutes the solvent composition was changed to 45 : 55 with linear increase. During the next one minute the solvent composition was reverted back to initial composition and the solvent mixture was held to equilibrate the column for 5 minutes. The total elution time was 20 minutes. Mycolic acid elutes between 10 and 13 minutes of run time and the chromatograms were visually compared.

### 2.6. Pattern Recognition

The chromatographic patterns of *M. tuberculosis *test isolates were visually matched with that of the chromatograms provided in the CDC manual. Reagent blank was treated as negative control to observe any sample contamination. Area under the peaks/cluster was used to calculate the effect of CPC on mycolic acid. Retention time is used to identify and validate the peaks of interest.

## 3. Results

### 3.1. FDA/EB and VC

**Figure 1:**
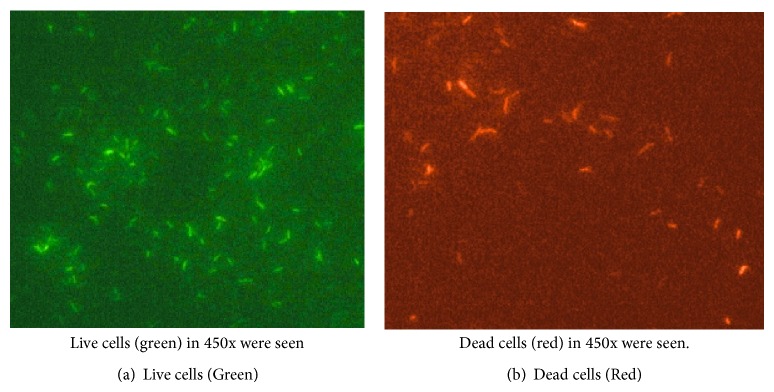
FDA-EB staining using Fluorescence microscopy.

FDA/EB consistently stained 70–90% of log phase cells as green (Figure 1(a)) and the remaining cells as red-orange. After treating the cells with CPC, 65–70% of the cells stained red-orange (Figure 1(b)). These results indicated that FDA entered the mycobacterial cells and hydrolyzed to fluorescein which accumulates intracellularly. These results also indicate that EB could penetrate the lipoid cell wall of the mycobacteria after cell membrane disruption by CPC treatment and intercalate with DNA. The stain also has an ability to penetrate clumps and stain the cells within the clumps. The metabolically active cells ranged from 60 to 90% on day zero in FDA-EB stain method. Proportion of metabolically active cells on day 7 reduced to 14 to 30% in controls and 7.5 to 40% in cells treated with CPC.

The mean log VC on day zero was 6.15. On day 7, the mean log VC in CPC untreated cells and in L-CPC and P-CPC treated cells was 5.92, 5.95, and 6.05, respectively (Table 1). The counts were comparable to zero-day controls.

### 3.2. HPLC

**Figure 2:**
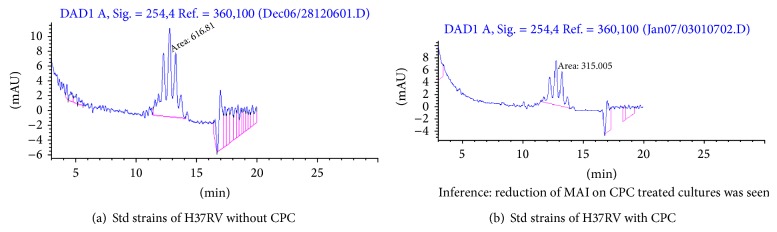
Determination of mycolic acid content of *M. tuberculosis* after exposure to P-CPC (H37RV).

**Figure 3:**
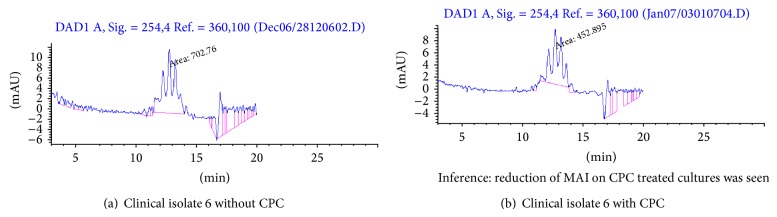
Determination of mycolic acid content of *M. tuberculosis* after exposure to P-CPC (clinical isolate).

The representative chromatogram of mycolic acid pattern for the standard strain (H37Rv) obtained from a control before and after CPC treatment was shown as Figures 2(a) and 2(b). Figures 3(a) and 3(b) showed representative chromatograms of mycolic acid obtained from clinical isolates before and after the addition of CPC. Mycolic acid content was reduced in *M. tuberculosis *when treated with CPC (Table 2). Synthesis of mycolic acids in mycobacteria was reduced when exposed to CPC due to the decreased metabolic activity of the organisms.

## 4. Discussion

The FDA-EB staining method is an alternative method of measuring mycobacterial viability. The feasibility of FDA-EB staining method for determining the viability of mycobacterial cells was investigated [5, 9–13]. The principle of FDA-EB staining method is that mycobacteria hydrolyze fluorescein diacetate to free fluorescein through nonspecific cellular esterase enzyme. Accumulation of fluorescein in metabolically active mycobacterial cells can be detected as green colored cells. Jarnagin and Luchsinger [9], while studying the viability of *M. tuberculosis*, observed a significant decrease in percentage of live bacteria stained as green upon increased periods of chemotherapy. Clinical strains included in this study were isolated sputum specimens from treatment naïve and follow-up patients. Though the isolates were taken from fresh log phase cultures, paucibacillary load in follow-up specimens is one of the factors for varying percentage of viability. This could also be one of the reasons for variation in percentage of viability in FDA-EB staining.

*M. tuberculosis *when not exposed to CPC possesses acetyl esterase and the cells are green in colour. When it was exposed to CPC, larger proportions were red in colour. These results showed that the cells become metabolically inactive. However, the metabolically inactive organisms grew well in LJ after removing the CPC from the culture. Results of viable count showed a minimal reduction after exposure to CPC. The viability of *M. tuberculosis *is reduced after 72 hours under ambient conditions [14]. The variation in one clinical isolate, number 3, may be due to the above reason. It is always possible that each clinical isolate behaves differently and it is unexpected. There could also be possibly technical error of using higher inoculums.

Mycolic acids are high molecular-weight, *λ*-alkyl-*β*-hydroxy fatty acids containing 70 to 90 carbon atoms. Paramasivan et al. [14] used HPLC analysis of mycolic acids to determine the sensitive and resistant strains after treating the cells with the antituberculosis drugs. Numbers of the peaks depicting different mycolic acids were same in CPC treated and untreated samples, but the area was reduced in CPC treated sample. The CPC reacts with the cell wall of the bacilli and cause damage. Mycolic acid content was shown to be reduced in HPLC analysis. This may be the reason for the reduction of AFB positives in smears stained by AP method, but viable count was not affected. The cell wall damage did not affect the growth and the damaged bacilli grew well on LJ medium. The difference in mycolic acid level between zero-day control and day 7-CPC treated strains indicates that there is decreased production of cell wall components. The cells though in live state do not multiply. CPC treated cells have pleomorphic morphology devoid of cell wall, which is depicted as reduction in mycolic acid index. These cells are however metabolically active as they regain their cell wall and grow when the pressure of CPC is removed. Thus all the above factors indicate that CPC may potentially act on the cell wall and degrade them. This was supported by the electron microscopy images that indicate CPC cells devoid of their outer cove, that is, cell wall (not shown in this paper).

The clinical strains are isolated from both treatment naive as well follow up patients. Hence, there could be difference in the load of bacilli among them which could be depicted in the mycolic acid index. Moreover, *M. tuberculosis *cells tend to vary in their morphology before and after exposure to Anti tuberculosis drugs. We have observed bacilli with reduction in their size especially after drug treatment. Hence, the amount of mycolic acid will also be less in these cells which could have been the cause for the decreased MAI before CPC treatment in these isolates. This could be one of the reasons for the low MAI even without treatment in two isolates (isolates nos. 5&6).

HPLC work was performed with P-CPC. Addition of bicarbonate to neutralize the alkaline conditions during extraction procedures reacted with L-CPC. The dark brown color from the reaction posed a hindrance during UV measurements in HPLC. The retention time of CPC and mycolic acid of *M. tuberculosis *was similar. Moreover, the elution of mycolic acid also became very difficult when using L-CPC rather that P-CPC.

## 5. Conclusion

The cells are metabolically inactive during storage with CPC but these cells grew well on LJ medium after removal of CPC. Viability of *M. tuberculosis *was maintained in CPC with minimal reduction. Mycolic acid content was also reduced when *M. tuberculosis *was treated with CPC for 7 days. This provides yet another evidence for the damage of cell wall of *M. tuberculosis *during CPC treatment.

## Figures and Tables

**Table 1:** Viable counts and proportion of metabolically active cells in control and CPC exposed cells of *M. tuberculosis*.

	0 day		Day 7					
Strains	Control		Control		L-CPC		P-CPC	
	VC	FDA∗	VC	FDA∗	VC	FDA∗	VC	FDA∗
Standard strain (H_37_RV)	6.27	90%	6.00	25%	6.17	22%	6.25	22%
Clinical isolate 1	6.25	80%	6.14	17%	5.90	25%	6.14	17%
Clinical isolate 2	5.84	80%	5.69	14%	5.84	7.5%	6.77	11%
Clinical isolate 3	6.20	70%	5.77	30%	5.90	20%	5.90	40%
Clinical isolate 4	6.07	60%	5.84	16%	5.84	20%	6.04	17%
Mean value (log_10_)	6.15		5.92		5.95		6.05	

FDA∗ expressed as percentage of green live cells present in the culture suspension.

**Table 2:** Mycolic acid index (MAI) values of clinical isolates.

Lab. no.	MAI without CPC	MAI with CPC
Standard H_37_Rv	816.81	315.005
Isolate 1	3283.01	835.877
Isolate 2	2945.36	1141.29
Isolate 3	1378.99	835.82
Isolate 4	1355.44	755.56
Isolate 5	580.531	294.499
Isolate 6	702.76	452.895

Minimum onefold decrease was observed in the CPC treated samples.
